# Correction: Mapping spatiotemporal distribution of forest carbon density in Xizang, China

**DOI:** 10.1371/journal.pone.0342180

**Published:** 2026-02-02

**Authors:** Li Cheng, Zi ling Yang, Yang yang Xia, Wen wen Guo, Rui qiang Ren, Jiang ping Fang

The authors, Zi ling Yang, Yang yang Xia, Wen wen Guo and Rui qiang Ren, should not have been attributed equal contribution to this work.
