# Supplementary material for: Mapping spatiotemporal distribution of forest carbon density in Xizang, China
Source: PLoS One. 2025 Sep 25;20(9):e0332890. doi: 10.1371/journal.pone.0332890 (PMC12463216; doi:10.1371/journal.pone.0332890)
Supplement: Attachment — Submitted filename: Review of distribution of forest carbon density in Tibet.pdf [file pone.0332890.s001.pdf]

Review of Mapping spatiotemporal distribution of forest carbon density in Tibet, China

Overall Recommendation: Major Revision

The manuscript presents a valuable contribution to regional carbon density estimation using machine learning and geospatial indices. The methodology is sound, the use of open-source tools is commendable, and the findings offer clear ecological and policy relevance. However, some **major/minor but important revisions** are necessary to enhance clarity, methodological justification and interpretation of results.

These include:

- Strengthening the justification for feature selection,

- Deepening the explanation of regional model performance,

- Expanding the discussion of limitations and future improvements.

Once these revisions are addressed, the manuscript will be **suitable for publication**.

Suggestions for Introduction Section

Line 45: Solid opening statement, but consider being more specific about what aspects of climate change (e.g., temperature rise, carbon imbalance) are being addressed.

Line 46: Good point, but "irreplaceable" could be rephrased to avoid exaggeration (e.g., "play a vital role").

Line 47: Slightly redundant — since it's known that trees are the main part of forests.

Line 48–49: Strong statement on services and carbon, but "thereby significantly affecting..." is a bit repetitive with earlier lines. Consider simplifying.

Line 50: "Will decrease" should be "decreased" (tense mismatch, you're referring to 2015–2020).

Line 51–52: Well positioned, though "self-evident" is subjective. Try rewording to “widely recognized.”

Line 53–54: Sentence is meaningful but wordy. Consider breaking into two for impact.

More clarity is needed on how carbon estimation differs between methods. Add examples or references for better clarity (e.g., which geostatistical model? which machine learning algorithm?). Define all acronyms clearly and early (e.g., GEF, SAR).

Missing Elements

i. A clear **research gap** and **novelty** statement.

ii. Rationale for using **Landsat 8** over other sensors like Sentinel-2.

iii. Short mention of **expected accuracy** or comparison with traditional methods.

iv. Clarify **how machine learning** is applied (brief method, like RF, SVM?).

Suggestion to study area Section

The section lacks ecological context; Tibet's role in carbon dynamics should be clearly stated. Climatic variation is described vaguely; use specific regions and quantify conditions. Long, complex sentences reduce clarity and need restructuring. Population and forest data are disconnected from the study focus explain their relevance to carbon estimation. Statistical claims lack citations, weakening credibility. The paragraph is descriptive but not analytical; link geographic and climatic features directly to the study's objectives.

Suggestion to Sample acquisition and statistical properties

Line 117: "From May to the end of June 2016..."

Please Specify why this time frame was chosen. Is it optimal for biomass measurement (e.g., growing season)? Also, mention whether the sampling was one-time or part of a long-term dataset.

Line 118: "total sampling area of 836,640 square kilometers..."

That's an extremely large area (nearly all of Tibet). Clarify if this refers to total area covered by all regions or the spatial distribution extent. Sampling intensity (e.g., per sq km) is unclear.

Line 119: "recorded the GPS coordinate information..."

What was the accuracy level of GPS devices used? Were coordinates collected in WGS84 or a different projection?

Lines 120–122: Good detail on DBH measurement standard. However, mention the tree selection criteria (e.g., random, dominant species, spacing), and how many trees were sampled per plot.

Lines 123–125: "...accurate to 0.1 cm... converted it into diameter."

Please Include potential sources of error here. Were double measurements or QA/QC checks performed?

Lines 126–127: Cleaning before measurement is good practice. You could add if saplings or trees below a certain DBH threshold were excluded.

Line 128: "forest carbon density was calculated in the laboratory..."

This is vague. What method was used (e.g., allometric equations, biomass expansion factors)? Was species-specific carbon fraction used?

Line 129: "Due to the wide distribution..."

Need Justification for choosing these five administrative regions should be added. Were ecological zones or forest types also considered?

Suggestion to Method of feature selection
Line 223 *"Given the high variability and potential nonlinear characteristics of forest carbon density distribution..."*
Suggestion: Briefly explain **why** forest carbon density is nonlinear (e.g., influenced by complex ecological interactions and environmental heterogeneity). Support with a scholarly reference.

Line 224–225 *"...we chose nonlinear Spearman correlation analysis..."*
Issue: A citation from Wikipedia is used.

Suggestion: Replace the Wikipedia citation with a **peer-reviewed journal** or **statistical textbook** for academic credibility.

Line 226–229 *"...we will comprehensively consider the correlation of each feature..."*
Issue: The term "features" is vague.
Suggestion: Clearly specify what features were considered (e.g., spectral bands, vegetation indices, topographic variables). Explain how many features were finally selected, and whether a **threshold correlation coefficient** was applied.Mention if **multicollinearity** between features was checked (important in machine learning).

Line 230 *"The experiment used three models..."*
Issue: Models are unnamed.
Suggestion: Specify which **three models** were used (e.g., Random Forest, XGBoost, SVR) and why they were chosen for comparison.

Line 231–233*"...using 5-fold cross-validation. Divide all datasets into five parts..."*
Cross-validation is appropriate.
Suggestion: Mention whether **stratified cross-validation** was used, especially if the data distribution is imbalanced. Clarify if a **random seed** was set for reproducibility.

Line 234–236 *"...evaluate the estimation performance... larger R^2, smaller RMSE and MAE."*

Good use of standard evaluation metrics.
Suggestion: Briefly define **R^2, RMSE, and MAE** to ensure clarity for all readers. State

whether performance was averaged across folds or if model performance varied significantly across regions.

Line 237–238 *"All algorithms and models… in Python 3.8 and MATLAB R2022b."*
Mentioning software is good practice.
Suggestion: Add **specific packages/libraries** used (e.g., scikit-learn, NumPy, MATLAB's Statistics Toolbox) for **reproducibility**.

3.1 Sample Statistical Properties

- High coefficient of variation (>1) confirms spatial heterogeneity.

- Non-normal distribution affects model assumptions.

- Lacks discussion of regional outliers or implications on modeling.

3.2 Feature Selection

- Smart integration of spectral (NDMI, Band3/4/7) and GEF features (CI, LSB, Geo).

- Geo chosen universally – reflects spatial dominance.

- Needs more statistical justification for feature choices.

3.3 Modelling Results

- XGB performs best overall, especially in Rikaze (R^2 = 0.965).

- RFR outperforms in Naqu – suggests local model–data fit matters.

- No mention of model complexity or training efficiency.

3.4 Feature Importance

- Geo is the dominant variable in most regions (>50% in Naqu).

- CI is consistently influential – links soil and carbon density.

- Good visuals, but regional differences need deeper exploration.

3.5 Dynamic Spatial Distribution

- Carbon density stable from 2016–2022; Linzhi and Shannan highest.

- Naqu remains lowest – clear ecological intervention target.

- Trend analysis is missing – no inter-annual change patterns.

4.1 Environmental Factor Response

- Explains role of climate and soil well – aligns with feature importance.

- Strong conceptual foundation.

- Could use more data-specific examples to connect theory to results.

4.2 Limitations and Caveats

- Acknowledges regional specificity and missing variables (e.g., microclimate).

- Transparency is strong.

- Should propose methods to integrate more diverse data.

4.3 Future Research and Applications

- Open-source tool offers practical value.

- Pushes for scaling and interdisciplinary work.

- Needs clarity on how tool will be shared, maintained, or validated globally.

5. Conclusion

- Models + GEF = strong prediction power.

- Geo and CI are core features.

- Solid wrap-up, but could briefly suggest satellite integration or future tech adoption.
