# Supplementary material for: Mapping spatiotemporal distribution of forest carbon density in Xizang, China
Source: PLoS One. 2025 Sep 25;20(9):e0332890. doi: 10.1371/journal.pone.0332890 (PMC12463216; doi:10.1371/journal.pone.0332890)
Supplement: Attachment — Submitted filename: Response to Reviewers.pdf [file pone.0332890.s002.pdf]

Response to Editorial Request

Manuscript ID: PONE-D-25-14912R1

Title: Mapping spatiotemporal distribution of forest carbon density in Tibet, China
Author: Dr. Li Cheng

Dear Editors,
Thank you for your detailed comments and for giving us the opportunity to revise our manuscript. We have carefully addressed the issue regarding the use of data from Google Earth Engine in Figures 1 and 6.

We confirm that:
Figure 1 has been revised using data from **USGS SRTMGL1 V003** for elevation (https://lpdaac.usgs.gov/products/srtmgl1v003/) and **Natural Earth** for administrative boundaries (https://www.naturalearthdata.com).
Figure 6 has been fully regenerated using **Landsat OLI/TIRS imagery** from the **NASA Landsat Visible Earth** archive (https://landsat.visibleearth.nasa.gov/), in place of data originally obtained through Google Earth Engine.

The remote sensing data used in Figure 6 were re-downloaded to match the same acquisition dates and sensor types as the original analysis. The results remain consistent due to the identical observation parameters.

In accordance with your request, we have also updated the **figure captions** to clearly state the sources of all data used, along with their public domain status or CC BY 4.0 compatibility.

We believe the current version of the manuscript fully complies with the PLOS ONE data and copyright policies. Thank you again for your guidance, and we appreciate your continued consideration of our work.

Sincerely,
Li Cheng, Ph.D.
